# Developing biases

**DOI:** 10.3389/fpsyg.2014.00634

**Published:** 2014-06-24

**Authors:** Ruben van de Vijver, Dinah Baer-Henney

**Affiliations:** Institut für Sprache und Information, Heinrich-Heine-Universität DüsseldorfDüsseldorf, Germany

**Keywords:** language acquisition, morphophonology, bias, voicing alternations, vowel alternations, production test

## Abstract

German nouns may alternate from singular to plural in two different ways. Some singular forms that end in a voiceless obstruent have a plural in which this obstruent is voiced. Another alternation concerns the vowel. Some singular forms with a back vowel have a plural form in which this back vowel is front. For each noun it has to be established individually whether it alternates or not. The voicing alternation is phonetically grounded, but the vowel alternation is not. Knowledge about such alternations involves two things. First, it involves knowledge of which words alternate and which words do not and second, it involves the ability to extend the alternations to novel words. We studied the knowledge of which words alternate and the proportion to which they alternate in two corpus studies. We studied the knowledge of speakers concerning which words alternate and what generalizations can be based upon these words by means of a production study. The production study involved words and nonces. We asked twenty 5 year-olds, twenty 7 year-olds, and ten adults to produce the plural for a given singular word and a plural for a given singular nonce. In the corpus study we found that both alternations occur with the same frequency. In the production of alternations in words we found that participants in all age groups make few mistakes. With respect to the production of alternations in nonce words, we found that the proportion of voicing alternations decreases with age, while the proportion of vowel alternations increases. We explain this change in the ability to generalize the alternations to nonces on the basis of the confidence speakers can have in a generalization. Young children have a small lexicon and they can form relatively unreliable generalizations on lexical distributions. They are, however, proficient users of language and have great phonetic experience. They can more confidently form generalizations on the basis of this experience. Adults have a large lexicon and, as a consequence, they can confidently form generalizations based on their lexicon. In addition, they know that many alternations are not based on phonetic considerations.

## 1. Introduction

The pronunciation of a word often varies with morphological context. Such variation is referred to as an alternation. In this paper we will focus on two alternations in German nouns. An example of the first alternation is provided by the singular and the plural of the word [bɛɐk] *mountain* (*Berg*). The singular ends in a voiceless obstruent, but this obstruent is pronounced as voiced in the plural [bɛɐgə] *mountains* (Berge). This alternation is referred to in this paper as a *voicing* alternation. An example of the second alternation is provided by the singular and the plural of the word [kuː] *cow* (*Kuh*). The back vowel in the singular corresponds to a front vowel in the plural: [kyːə] *cows* (*Kühe*). This alternation is referred to in this paper as a vowel alternation. Both alternations are unpredictable in the sense that one needs to know whether or not a word alternates; many words have no alternation^1^.

The voicing and the vowel alternation differ in their phonetic grounding. The voicing alternation has a phonetic motivation. A voicing contrast is difficult to perceive word-finally (Steriade, 1997) and voiced obstruents are easier to produce between sonorants than voiceless obstruents (Westbury and Keating, 1986). The vowel alternation is not phonetically motivated in contemporary German. It is fossilized from a vowel harmony process that is no longer productive in German (Klein, 2000).

Native speakers have knowledge of such alternations in two different ways. The first aspect of such knowledge concerns knowledge of alternations in words. If native speakers know a singular and the corresponding plural they have knowledge of this alternation. The second aspect of this knowledge involves the ability to generalize an alternation to novel words. This latter aspect of the knowledge of a native speaker goes beyond knowing just a list of words and suggests that native speakers have knowledge of relations among words (Pierrehumbert, 2000).

In order to generalize alternations to novel words, speakers may rely on different sources of information. One important source of information is frequency in the input (Bybee, 2001a,b, 2006, 2007) and frequency in the lexicon of a speaker (Pierrehumbert, 2006). Pierrehumbert found that velar softening in English—alternations such as found in the pair [əlɛktrIk] ~ [əlɛktrisIti]—are produced rarely in nonces, and that their production depends on the knowledge of many latinate words by the participant. Buckler (2014) investigates the acquisition of voicing alternations in Dutch and German and she finds that German children are able to recognize a voicing alternation at 9 months of age, but Dutch children do not. She also counted the frequency of the voicing alternations in both languages and finds that it is more frequent in German than in Dutch. Her finding indicates that frequency is indeed an important factor in the acquisition of alternations. In order to achieve knowledge about which words alternate the learner needs to acquire a lexicon from the input—the surrounding language. Once learners have acquired a list of words they can form generalizations over these.

Another important source of information is the phonetic grounding of an alternation. If an alternation facilitates the pronunciation or the perception of a word it may be easier to generalize it to novel items than when an alternation does not improve production or perception (Hayes and Steriade, 2004; Hayes and Wilson, 2008; Baer-Henney and van de Vijver, 2012). Baer-Henney and van de Vijver study the influence of frequency and phonetic grounding on the acquisition of morphophonological alternations by Germans in using an artificial language learning experiment. Baer-Henney and van de Vijver (2012) created an artificial language in which the vowel of the plural suffix harmonized with the vowel of the stem. The suffix harmonized for the feature [back] in one language and in the other language the backness of the suffix vowel was associated with the feature [lax] of the stem vowel. The first alternation is phonetically grounded, but the second alternation is not. The alternation in each language was provided as input to the learner in two frequency conditions. In one condition the plurals were 50% of the total amount of input and in another frequency condition the plurals made up 25% of the total amount of input. This created four different artificial languages, a frequent and an infrequent backness language and a frequent and an infrequent laxness language. After a phase with only input, participants were asked to produce a plural. In the frequent condition both the backness alternation and the laxness alternation was learned. In the infrequent condition the backness alternation was generalized more often than the laxness alternation. This findings suggests that, in addition to a frequency bias, there is also a bias for phonetically based alternations. What is not known, however, is whether these biases exert an equal influence at all points in the acquisition of a language.

One reason these biases—one for frequency in the speaker's lexicon and one for the phonetic grounding of an alternation—may develop over time is that the confidence in these biases may change over time. It is reasonable to suppose that a learner relies on a bias in proportion to the amount of confidence it inspires. Generalizations in which she can place greater confidence are preferred in comparison to generalizations in which it can have less confidence (Mikheev, 1997); see also Albright and Hayes (2003) and Pierrehumbert (2006). One source of confidence in generalizations is frequency. If a particular pairing occurs very frequently and is drawn from a large pool of samples it is predicted to lead to a generalization in which it can have great confidence. If, on the other hand, such pairings come from a small sample the cognitive system can have less confidence in the generalization. There is a difference between a pairing that occurs in 1 case out of a sample of 5 and a pairing that occurs in 100 cases out of a sample of 500 (Mikheev, 1997; Albright and Hayes, 2003; Pierrehumbert, 2006). The influence of frequency on generalizations of alternations is uncontroversial, but it is not clear whether the influence is the same for all age groups. Children have a relatively small lexicon and may therefore be skeptical about generalizations based on lexical frequency alone in comparison to adults. They are, however, experienced speakers and listeners. They can use this experience to derive generalizations. Children are, however, experienced speakers and listeners and consequently they rely more on their knowledge of speaking and listening in order to form a generalization.

We are now in a position to formulate our hypotheses. In order to learn about alternations children need to know a number of words with an alternation. We expect that, if this is the case, this is reflected in their production of such alternations in words. If this is true, we expect that children use this information to form generalizations about the alternations that they can apply to nonces. If in the input to the children the alternations are evenly distributed and this is their primary source of information for generalizations then we expect that in the production of alternations in nonces the proportions found in the input will be reflected.

If, on the other hand, the input frequency is not the sole determinant of the generalizations and that the evidence is weighted on the basis of the amount of certainty it provides, we expect that children place more confidence in the phonetic grounding of an alternation than adults. Children have a small lexicon and, therefore, place relatively little confidence in generalizations that are based on their lexicon. As they are proficient speakers and hearers they place more confidence in generalizations that reflect their knowledge of phonetics. We therefore expect that children, who have a small lexicon, may overestimate the proportion of voicing alternations—which are phonetically grounded—and underestimate the proportion of vowel alternations—which are not—both in comparison to adults.

## 2. Materials and methods

### 2.1. Estimating the frequency of voicing and vowel alternations in nouns

We first present the analysis of our corpora, since one of them served as basis for the creation of our nonces and it served as an estimate of the proportion of voicing and vowel alternations in the input of the children and adults.

We created two corpora in order to estimate the proportion of each alternation in the input. We restricted ourselves to nouns, since children appear to track frequencies per part of speech (Berko-Gleason, 1958). We counted types rather than tokens, since type frequencies is what adults track (Ernestus and Baayen, 2003).

One corpus consists of all 945 singular–plural pairs taken from a corpus based on data from the national newspaper *Frankfurter Rundschau*^2^. The other corpus consists of all 345 singular–plural nouns taken from the Simone-corpus, which can be found on CHILDES (MacWhinney, 2000). We only used the child-directed speech from the Simone-corpus and, in addition to studying the frequency of the alternations, this phonotactics of the words in this corpus served as a basis for the phonotactics of the nonces (see **Table 3**).

The proportions of the type alternations are the same in both corpora (for voicing alternation: Fisher's Exact Test *p* = 0.5, odds ratio = 1.2, 95% confidence interval = 0.69–2.04,; for vowel alternation: Fisher's Exact Test: *p* = 0.1, odds ratio = 1.33, 95% confidence interval = 0.93–1.89). The raw numbers are given in Tables 1, 2, in which the alternation contexts refer to the number of words ending in an obstruent for the voicing alternation and the number of words with a back stem vowel for the vowel alternation.

**Table 1:** **Voicing alternation and vowel alternation in the Frankfurter Rundschau corpus (types)**.

	**Alternation contexts**	**Actual alternations**	**Proportion (%)**
Voicing	270	59	21.8
Vowel	457	104	22.8

**Table 2:** **Voicing alternation and vowel alternation in the child-directed speech corpus (types)**.

	**Alternation contexts**	**Actual alternations**	**Proportion (%)**
Voicing	103	27	26.2
Vowel	234	71	30.3

The frequency of both alternations in nouns is comparable. In the input children and adults are as likely to encounter a voicing alternation as a vowel alternation. This suggests that, as to the words of the children and adults, they have an equal chance of learning about voicing alternations as about vowel alternations.

### 2.2. Material

We used 24 words and created 39 nonces—phonotactically legal words that do not exist in German—in a production test (Berko-Gleason, 1958) in which we presented the participants with an item in the singular and asked them to provide the plural.

The words are common words, taken from a list of words that 2-year-olds are supposed to know (Grimm and Doil, 2000) and a few words that are part of Caroline corpus in CHILDES (MacWhinney, 2000) which Caroline used at an early age. Eight of the words ended in an obstruent, four with a voicing alternation and four without. Another eight of the words had a back vowel, four with a vowel alternation and four without. The last batch of eight words had a back vowel and a final obstruent, four of which had both a vowel and a voicing alternation and the other four had no alternation. The full list of words is given in section A.1.

To create our nonces words we extracted a corpus of 398 singular–plural pairs from a child-directed speech corpus [the Caroline corpus (MacWhinney, 2000)] and analyzed the pairs phonotactically. We wanted to ensure that the nonces resembled words, since such nonces are rated as better examples of words and are treated more like words (Frisch et al., 2000; Friedrich and Friederici, 2005). By basing ourselves on the rhymes of a corpus of child-directed speech we ensured that the rhymes of our nonces resembled the rhymes of words used in addressing children. The distribution of environments is given in Table 3; the gap in the table concerns nonces that would not have the environment for a voicing alternation nor for a vowel alternation; they would thus fall beyond the scope of our study. The full list of nonces is given in section A.2.

**Table 3:** **Phonotactics of the rhymes of the nonces**.

**Voicing alternation context**			
**Vowel alternation context**		**No vowel alternation context**	
Cluster	No cluster	Cluster	No cluster
Four items	Six items	Two items	Three items
[dant]	[bɔt]	[fɛns]	[meːk]
**No voicing alternation context**			
**Vowel alternation context**			
Cluster	No cluster		
11 items	13 items		
[dɔɐm]	[moːl]		

### 2.3. Participants

We tested three groups of participants: Twenty 5-year-olds (mean age 4;9.11), twenty 7-year-olds (mean age 7;1.10), and twenty adults (mean age 29;11). They were all from the area around Potsdam, Germany, and monolingual native speakers of German.

### 2.4. Procedure

The participants were seated in front of a computer. We told them that they would see pictures of familiar and unknown items on the monitor.

In a short practice session the participant was shown pictures of an apple *Apfel* [a$\overset{⌢}{\text{pf}}$əl] (*Apfel*), the moon *Mond* [moːnt] (*Mond*), a forest *Wald* [valt] (*Wald*) and, as an example of a nonce, a fantasy animal, a wug [vak]. After each picture the participant was prompted to provide the plural. If the participant did not provide any, the experimenter provided the plural. This was repeated until the participant provided the plural.

In the test phase the experimenter told the participant what was shown on the screen, for example, “Look, a [gɔɐp].” (“Guck mal, ein [gɔɐp].”). Then a picture with two gɔɐps appeared and the experimenter said “Look, now there are two! There are two…?” (“Guck mal, jetzt sind da zwei. Das sind zwei…?”) thus prompting the participant to provide the plural. Each participant was tested on 39 nonces and 24 words presented in a different, random order.

The whole session was recorded and transcribed by the experimenter and independently by the second author by auditory inspection and visual inspection. In almost all cases the raters agreed in their judgment. In the few cases where the transcribers disagreed the first author transcribed the target word blindly—without being given any information about what word or nonce was intended. In all cases it could be determined what the child had said by at least two of three transcribers.

### 2.5. Results

Before we discuss the results for the nonces we will briefly discuss the results for the words. The task was a production task in which the participants were free to give whatever answer as plural. In most cases the answers contained some modification of the singular we provided them with—adding a suffix or changing the vowel—but in some cases the participants did not change anything. Five year-olds produced a total of 478 responses, of which 385 (80%) had a change to the singular we provided them with and 93 (20%) words were a repetition of the singular. Seven year-olds produced 480 responses, 470 of which (97%) contained some kind of modification and 10 cases were repetitions of the singular we provided them with. Adults provided us with 479 responses, 477 (99.5%) of which contained some kind of modification and 2 (0.5%) were repetitions of the singular we provided them with. Only those responses that had any modification provide us with information about alternations, so we only included these responses in our analysis of the production of words^3^.

Excluding the bare nouns, the 5 year-olds produced plurals for 131 items that require voicing, such as [$\overset{⌢}{\text{pf}}$eɐt] *horse* (*Pferd*) which has the plural [$\overset{⌢}{\text{pf}}$eɐdə] (*Pferde*). They correctly produced voicing alternations in 127 words (97%) and failed to produce it in 4 words (3%). There were 126 words that end in an obstruent, but that do not require a voicing alternation, such as [flɛk] *stain* (*Fleck*), which has as plural [flɛkən] (*Flecken*). In 11 of these (9%) the children produced a voicing alternation. There were 126 words that require a vowel alternation in the plural, such as [kuː] *cow* (*Kuh*) which has as plural [kyːə] (*Kühe*). In 16 of these (13%) they failed to produce a vowel alternation. There were 127 words with a back vowel in the singular that do not require a vowel alternation in the plural, such as [ʃuː] *shoe* (*Schuh*) which has as plural [ʃuːə] (*Schuhe*). In 16 of these (13%) the children produced a vowel alternation.

As for the 7 year-olds, again excluding the bare nouns, we analyzed the errors in the way. There were 160 singulars that end in an obstruent, the plural of which has a voiced obstruent. Of these, 157 (98%) were correctly voiced and in three words (2%) they produced no voicing alternation. There were 151 words that ended in an obstruent which do not have a plural with a voicing alternation. Of these, 5 words (3%) were erroneously pronounced with a voicing alternation. There were 160 words that require a vowel alternation in the plural and in one word (0.4%) the vowel alternation was not pronounced. There were 153 words that do not require a vowel alternation, 15 of which (10%) were erroneously produced with a vowel alternation.

The adults produced all plurals correctly.

In short, as to the words, all groups of participants know the words very well. All age groups make few mistakes and the numbers of mistakes decrease with age. Their knowledge of alternations in words provides all age groups with the basis for generalizations which can be applied to produce alternations in nonces.

Now, let us turn to the results of the nonces.

As with the words the participants sometimes repeated the nonce without any change; the plural they provided was identical to the singular we presented them with. This tendency was stronger in younger children than in adults. Five-year-olds answered with a bare stem in 538 cases (69%) and with an inflected form in 242 cases (31%). Seven-year-olds answered with a bare stem in 301 cases (39%) and with an inflected form in 477 cases (61%) and adults answered with a bare stem in 95 cases (12%) and with an inflected form in 685 cases (88%). In our analysis we included only those answers that could, in principle, be identified as a plural.

Table 4 shows that the amount of voicing alternations produced in nonces decreases with age. Five-year-olds produced 32% voicing alternations, seven-year-olds produced 21.4% voicing alternations and adults produced 16.9% voicing alternations.

**Table 4:** **Nonces: voicing alternations across age groups**.

	**Alternation (%)**	**No alternation (%)**
Five-year-olds	43 (32)	91 (67.9)
Seven-year-olds	45 (21.4)	165 (78.5)
Adults	49 (12)	240 (88)

Table 5 shows that the amount of vowel alternations increases with age. Five-year-olds produced 1.6% vowel alternations, seven-year-olds produced 5.1% vowel alternations and adults produced 10.8% vowel alternations. This is summarized in Table 5.

**Table 5:** **Nonces: vowel alternations across age groups**.

	**Alternation (%)**	**No alternation (%)**
Five-year-olds	11 (1.6)	669 (98.7)
Seven-year-olds	35 (5.1)	645 (94.8)
Adults	74 (10.8)	606 (89.1)

**Figure 1:**
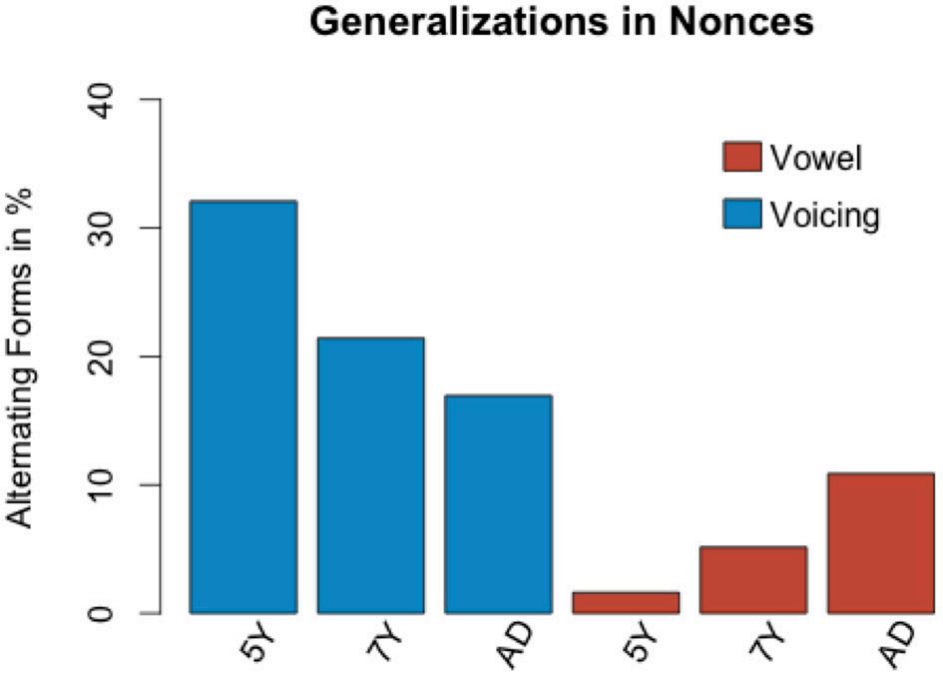
**The proportion of voicing alternations decreases with age**. The proportion of vowel alternations increases with age.

A graphical overview of these proportions of all alternations produced in nonces is shown in Figure 1.

We calculated the maximum likelihood of the proportion of voicing alternations for all three populations and the associated 95% confidence intervals based on a simulation of 5000 repetitions of our experiments (Gelman and Hill, 2007). We ran this analysis because the data contained too few cases to run a binomial regression analysis.

**Figure 2:**
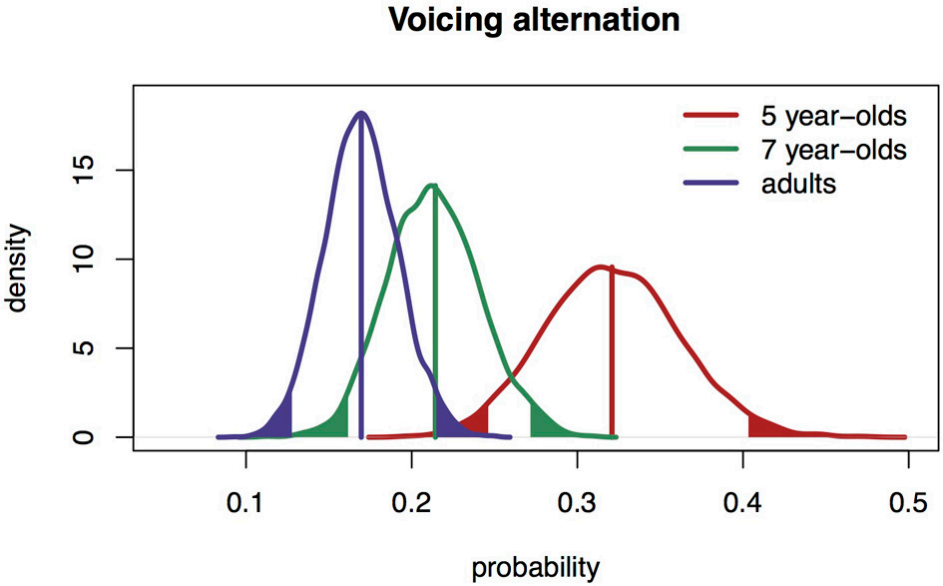
**The maximum likelihood distribution of the proportions of voicing alternations with 95% confidence intervals (solid bands) for 5-year-olds, 7-year-olds, and adults**.

Each bell curve shows the expected distribution of the proportion of alternations for a population (Gelman and Hill, 2007). It can be seen that the distributions of the 5-year-olds and the adults do not overlap. The 5-year-olds produce more voicing alternations than the adults. The distribution of the 7-year-olds is between the distribution of the 5-year-olds and adults. This is illustrated in Figure 2.

**Figure 3:**
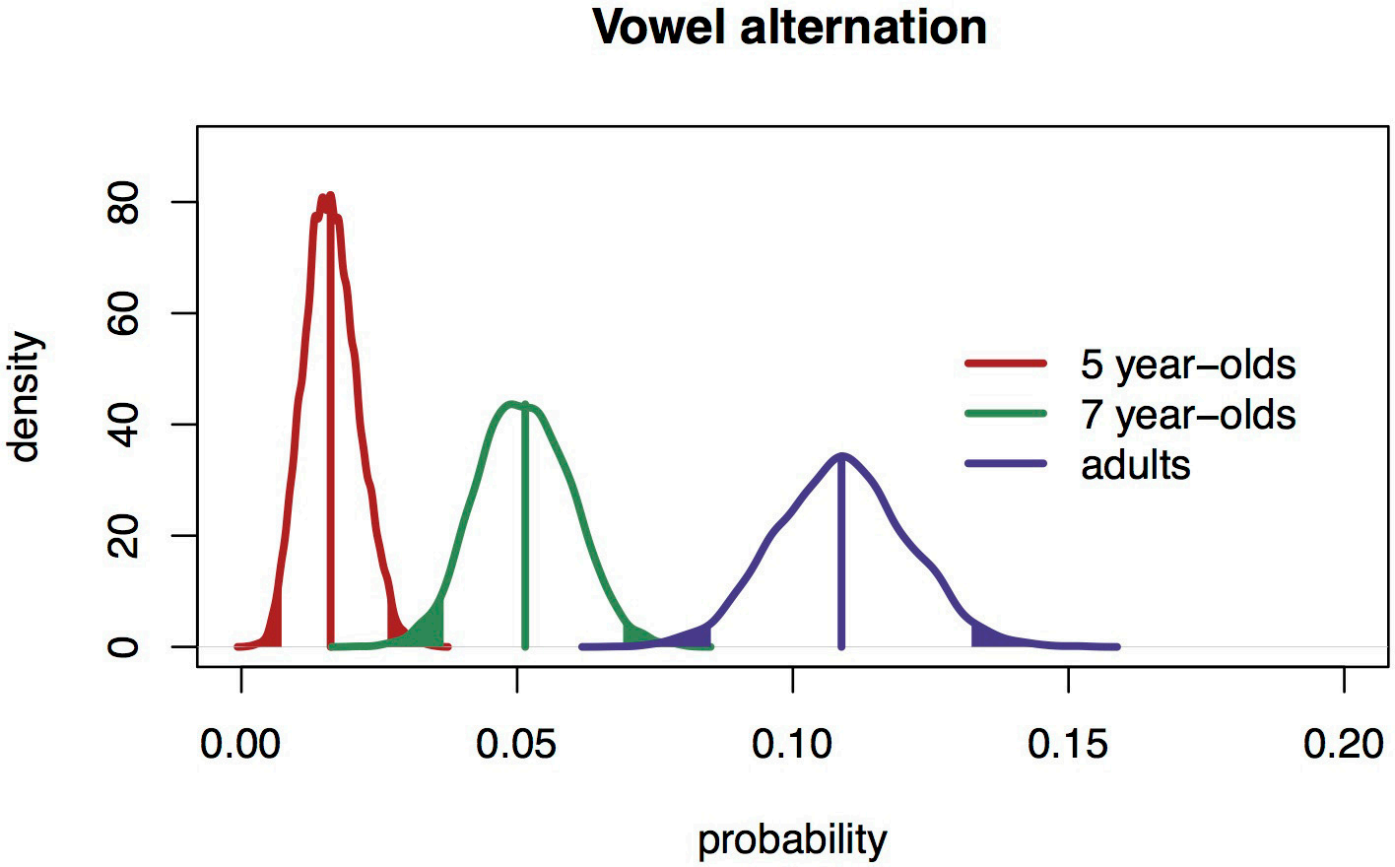
**The maximum likelihood distribution of the proportions of vowel alternations with 95% confidence intervals (solid bands) for 5-year-olds, 7-year-olds, and adults**.

The maximum likelihood distribution of the proportion of vowel alternations for all three populations and the associated 95% confidence intervals, also based on 5000 repetitions of our experiment is shown in Figure 3. This distribution shows that adults produce more alternations than 5-year-olds. Seven-year-olds produce a proportion that is between the proportions of five-year-olds and adults.

## 3. Discussion

We ran a production experiment in order to study the development of generalizations concerning voicing and vowel alternations in German nouns. We tested twenty 5-year-olds, twenty 7-year-olds, and twenty adults. In addition, we also studied the proportion of voicing and vowel alternations in nouns. It turns out that in two corpora the proportion of voicing and vowel alternations is the same.

Given the frequency of the alternations in nouns in the input (see Tables 1, 2) and both the correct productions in words and the error patterns in words one might have expected that both alternations are extended to nonces at the same rate. Both 5-year-olds and 7-year-olds produce voicing and vowel alternations in words largely correctly and, as for their errors, both groups of participants overgeneralize the alternations to the same extent to novel words and they both fail to produce required alternations to the same extent. We found that all participant groups extended voicing and vowel alternations to nonces. With increasing age the proportion of voicing alternations falls off, while the proportion of vowel alternations climbs.

In the light of the results of our production experiment concerning words, this is unexpected. The children know both alternations in words well—which provides them with a basis for generalizations—and in their input both alternations occur with the same frequency. If the distribution of the alternations in the input is the sole source of information they use we would have expected that both are produced with the same proportion in the nonces. This is clearly not the case.

We explain this as follows. Children have evidence for pairs that alternate and pairs that do not; both in words with voicing alternations and in words with vowel alternations. It is, therefore, impossible to find a generalization which can be completely trusted. Since a 5-year-old has a relatively small lexicon any generalization based on their lexicon is necessarily based on a relatively small sample and comes, consequently, with a high degree of uncertainty. However, the 5-year-old is an experienced language user. Generalizations that are based on phonetic grounding are made with a fair amount of confidence. As a consequence, they will be more confident that a voicing alternation is warranted, as this alternation is found in the words they know, which, however, inspires little confidence, and such an alternation is phonetically grounded, which inspires much more confidence. They will have little confidence in extending vowel alternations to nonces, even though such alternations occur and in their lexicon, since they are uncertain concerning generalizations based on their lexicon and this alternation is not supported phonetically.

Seven-year-olds have a larger lexicon than five-year-olds. They have noticed that the proportion of voicing alternations is not as large as they assumed when they were five and that the proportion of vowel alternations is larger than they assumed when they were five. These insights are based on a larger sample than when they were five and, therefore, they are confident that their generalizations reflect the proportions found in their lexicon. Since their lexicon is larger it provides a more secure basis for their generalizations and they can rely less on generalization based on phonetic grounding.

Adults, of course, have the best sample of all: A large lexicon. They can be very confident that their lexicon serves as a basis for their generalizations. They can almost completely ignore any further information that derives from substance as being unreliable. This explains why in many experiments adults reflect the lexical proportions in inflections of novel words (Ernestus and Baayen, 2003), leaving only very little evidence for the presence of a bias for substantively based alternations (Albright and Hayes, 2003; Zhang and Lai, 2008, 2010; Hayes et al., 2009; Zuraw, 2010). It is interesting that the proportion of both alternations produced by adults in the nonces is similar and that the proportion of both alternations in the corpora is also the same. The fact that the absolute proportions in the data of the corpora and in the production of alternations in the nonces is different is probably a result of the fact that the phonotactics of the nonces are a subset of the phonotactics of the words represented in the corpora.

This finding is in agreement with findings in artificial language experiments. In such experiments, where there is no lexical support, adults often show biases toward substantively based generalizations (Wilson, 2006; Finley and Badecker, 2009; Baer-Henney and van de Vijver, 2012). As their lexicon cannot serve as a secure basis for their generalizations they rely on another source of information for confidence in their generalizations: phonetic grounding.

These results can be formalized in several ways, provided the theory is able to incorporate information about frequency and is able to slightly adjust this frequency on the basis of the strength of the evidence. One formal model in which the results can be explained is the Minimal Generalized Learner proposed by Albright and Hayes (2003). In this model generalizations are the result of a comparison of two forms, for example, a singular form and a plural form. The learner takes a singular form, such as [vɛɐk] *factory, work, opus* (*Werk*) and its plural [vɛɐkə] (*Werke*) and compares them. In doing so, the learner concludes that forming a plural consists of adding [ə] to [vɛɐk]. The learner will encounter other pairs. For example, it will encounter the singular [bɛɐk] *mountain* (*Berg*) and the plural [bɛɐgə] (*Berge*). Here the learner will conclude that the first three segments [bɛɐ] remain stable over both forms and that the [k] of the singular changes to [gə] in the plural. In the case of the pair [bɑl] *ball* (*Ball*) and [bɛlə] (*Bälle*) the rule will be that the back vowel of the singular corresponds to a front vowel in the plural and that a schwa is added. In this way the learner compares all pairs it encounters and forms rules—generalizations—that map singular forms onto plural forms. The rules themselves can be further generalized over. For example, once the learner encounters to pair [tɑk] *day* (*Tag*) and [tɑgə] (*Tage*) it will be able to use this rule and compare it to the rule for [bɛɐk] ~ [bɛrgə]. The learner will notice the similarities and generalize that a singular form that ends in a dorsal voiceless stop preceded by a low vowel corresponds to a voiced dorsal stop followed by a schwa in the plural. The more pairs are captured by the rules the more confidence is placed in it and the greater the weight of the rule (Albright and Hayes, 2003). This ensures that lexical frequencies are tracked by the learner. In short, the larger the lexicon the greater the confidence in the rules. In addition the weight of the rules can be adjusted by taking into account the phonetic groundedness of a rule and giving those rules a greater weight that facilitate production or perception of the output (Wilson, 2006). The confidence placed in this additional weight is relative to the general confidence in the rules; the smaller the general confidence in the rules the larger the weight of phonetic groundedness. This interpretation agrees with experimental results on biases for phonetic groundedness (Wilson, 2006; Baer-Henney and van de Vijver, 2012) and experimental results concerning the ability to track lexical frequencies (Ernestus and Baayen, 2003). When learners have no other evidence but their knowledge of phonetics, such as in artificial language experiments, they tend to rely more on phonetic information, but if they can rely on lexical frequencies, as in nonce word productions, they will prefer that source of information.

## Funding

This research was supported by German Research Council Grant No. VI 223\2-1 “The acquisition of voicing and vowel alternations in German morphophonology” to Ruben van de Vijver. The project is part of the German Research Council Priority Program 1234: “Phonological and phonetic competence: between grammar, signal processing, and neural activity.”

### Conflict of interest statement

The authors declare that the research was conducted in the absence of any commercial or financial relationships that could be construed as a potential conflict of interest.

## Footnotes

^1^The acquisition of suffixes—including vowel alternations—has received attention in the literature but this strand of research ignores voicing alternations (Köpcke, 1988; Clahsen et al., 1992; Kauschke et al., 2011).

^2^The corpus was extracted with the assistance of Gerlof Bouma.

^3^For an overview of the suffixes produced see van de Vijver and Baer-Henney (2013); in that paper there is no evidence that the suffix [s] is treated as a default. Rather its distribution is very similar to other reports in the literature (Kauschke et al., 2011) and seems to follow its lexical distribution.

## Appendix

### A.1 WORDS

**Table:**

**Singular**	**IPA**	**Plural**	**IPA**	**Gloss**
Ball	[bal]	Bälle	[bɛlə]	ball
Baum	[baʊm]	Bäume	[bɔYmə]	tree
Bett	[bɛt]	Betten	[bɛtən]	bed
Bild	[bIlt]	Bilder	[bIldəɐ]	picture
Brot	[broːt]	Brote	[broːtə]	bread
Bus	[bʊs]	Busse	[bʊsə]	bus
Elefant	[eːləfant]	Elefanten	[eːləfantən]	elephant
Fahrrad	[faːraːt]	Fahrräder	[faːreːdəɐ]	bicycle
Fleck	[flɛk]	Flecken	[flɛkən]	stain
Hand	[hant]	Hände	[hɛndə]	hand
Haus	[haʊs]	Häuser	[hɔYzəɐ]	house
Katze	[ka$\overset{⌢}{\text{ts}}$ə]	Katzen	[ka$\overset{⌢}{\text{ts}}$ən]	cat
Kind	[kInt]	Kinder	[kIndəɐ]	child
Kleid	[klaIt]	Kleider	[klaIdəɐ]	dress
Kuh	[kuː]	Kühe	[kyːə]	cow
Licht	[lIçt]	Lichter	[lIçtəɐ]	light
Maus	[maʊs]	Mäuse	[mɔYzə]	mouse
Ohr	[oːɐ]	Ohren	[oːrən]	ear
Pferd	[$\overset{⌢}{\text{pf}}$ɛɐt]	Pferde	[$\overset{⌢}{\text{pf}}$ɛɐdə]	horse
Puppe	[pʊpə]	Puppen	[pʊpən]	doll
Schaf	[ʃaːf]	Schafe	[ʃaːfə]	sheep
Schiff	[ʃIf]	Schiffe	[ʃIfə]	boat
Schuh	[ʃu]	Schuhe	[ʃuːə]	shoe
Stuhl	[ʃtuːl]	Stühle	[ʃtyːlə]	chair

### A.2 NONCES

**Table:**

IPA
[bans]
[bɔt]
[dɑnt]
[daʊl]
[dInt]
[doːtɐ]
[dɔɐm]
[dʊs]
[fam]
[fɛns]
[gal]
[gɔɐp]
[guːl]
[gʊmɐ]
[kaʊs]
[koːn]
[koːpɐ]
[laːzɐ]
[lʊm]
[marɐ]
[meːk]
[moːl]
[naːkɐ]
[nuːfɐ]
[paʊfɐ]
[pɑIt]
[pɔn]
[kvasɐ]
[rʊlɐ]
[zaːɐ]
[ʃʊnt]
[zɔɐnɐ]
[zuk]
[taːf]
[taʊlɐ]
[tIs]
[tʊŋ]
[vɔkɐ]
[voːt]
